# Exploring the physiologic role of human gastroesophageal reflux by analyzing time‐series data from 24‐h gastric and esophageal pH recordings

**DOI:** 10.14814/phy2.12051

**Published:** 2014-07-17

**Authors:** Luo Lu, John C. Mu, Sheldon Sloan, Philip B. Miner, Jerry D. Gardner

**Affiliations:** 1 Department of Statistics, Stanford University, Stanford, 94305, California; 2 Department of Electrical Engineering, Stanford University, Stanford, 94305, California; 3 Janssen Research and Development, Titusville, 08560, New Jersey; 4 Oklahoma Foundation for Digestive Research, University of Oklahoma Health Sciences Center, Oklahoma City, 73104, Oklahoma; 5 Science for Organizations, Inc., Mill Valley, 94941, California

**Keywords:** Esophageal pH, gastric pH, gastroesophageal reflux, vector autoregression

## Abstract

Our previous finding of a fractal pattern for gastric pH and esophageal pH plus the statistical association of sequential pH values for up to 2 h led to our hypothesis that the fractal pattern encodes information regarding gastric acidity and that depending on the value of gastric acidity, the esophagus can signal the stomach to alter gastric acidity by influencing gastric secretion of acid or bicarbonate. Under our hypothesis values of gastric pH should provide information regarding values of esophageal pH and vice versa. We used vector autoregression, a theory‐free set of inter‐related linear regressions used to measure relationships that can change over time, to analyze data from 24‐h recordings of gastric pH and esophageal pH. We found that in pH records from normal subjects, as well as from subjects with gastroesophageal reflux disease alone and after treatment with a proton pump inhibitor, gastric pH values provided important information regarding subsequent values of esophageal pH and values of esophageal pH provided important information regarding subsequent values of gastric pH. The ability of gastric pH and esophageal pH to provide information regarding subsequent values of each other was reduced in subjects with gastroesophageal reflux disease compared to normal subjects. Our findings are consistent with the hypothesis that depending on the value of gastric acidity, the esophagus can signal the stomach to alter gastric acidity, and that this ability is impaired in subjects with gastroesophageal reflux disease.

## Abstract (precis)

We found that in pH records from normal subjects, as well as from subjects with gastroesophageal reflux disease alone and after treatment with a proton pump inhibitor, gastric pH values provided important information regarding subsequent values of esophageal pH and values of esophageal pH provided important information regarding subsequent values of gastric pH. Our findings are consistent with the hypothesis that depending on the value of gastric acidity, the esophagus can signal the stomach to alter gastric acidity, and that this ability is impaired in subjects with gastroesophageal reflux disease.

## Introduction

Previously, we found that in normal subjects, subjects with gastroesophageal reflux disease (GERD) and subjects with GERD treated with a proton pump inhibitor (GERD + PPI), both gastric pH and esophageal pH reflect an underlying stochastic process that generates a fractal pattern over time (Gardner et al. 2005). The fractal pattern of gastric pH and esophageal pH plus the statistical association of sequential pH values for up to 2 h led to our hypothesis that the fractal pattern of pH values encodes information regarding gastric acidity and that depending on the value of gastric acidity, gastroesophageal reflux causes the esophagus to signal the stomach to alter gastric acidity by influencing gastric secretion of acid or bicarbonate (Gardner et al. 2005). This hypothesis was supported by the subsequent finding that in normal subjects infusing acid into the esophagus, but not into the stomach, reduced gastric acidity (Blondeau et al. 2009).

Infusing acid into the esophagus of normal subjects, however, did not alter gastric acidity during fasting and although the magnitude of the decrease in gastric acidity varied directly with meal‐stimulated gastric acid secretion, the decrease in gastric acidity could only be detected beginning 3 h after the end of the meal (Blondeau et al. 2009). Even though results from our infusion study were consistent with the hypothesis that esophageal acidity can influence gastric acidity, perhaps infusing acid into the esophagus at a constant rate and concentration might be too far removed from the typical physiologic setting to provide a realistic test of our hypothesis.

Under our hypothesis values of gastric pH should provide information regarding values of esophageal pH and vice versa. To examine possible relationships between gastric pH and esophageal pH over time, we used vector autoregression (VAR) to analyze data from 24‐h recordings of gastric pH and esophageal pH. VAR is a theory‐free set of inter‐related linear regressions used to measure relationships that can change over time. VAR was originally developed and used subsequently to analyze macroeconomic data (Sims 1980). The most common use of VAR for human data appears to be to analyze time‐series data from neurophysiologic experiments (e.g., (Chen et al. 2011)). VAR has also been used to analyze time‐series data from a variety of fields including ecology (Dhoray and Teelucksingh 2007), epidemiology (Hii et al. 2012), public health (Joyce and Grossman 1990; Langley et al. 2012), clinical medicine (Tschacher and Kupper 2002; Tsacher et al. 2003; Jones et al. 2008; Rosmalen et al. 2012; Bringmann et al. 2013), studies of human migration (Gorbey et al. 1999), models of gene regulatory networks (Lim et al. 2013), prediction of disease biomarkers (Rochon 2003), developing predictors of respiratory motion in robotic surgery (Ernst et al. 2013), and relating blood pressure to heart rate (Matsukawa and Wada 1997).

In the present analyses, we have explored whether sequential values of gastric pH provide important information about subsequent values of esophageal pH as well as whether sequential values of esophageal pH provide important information about subsequent values of gastric pH. “Important information” means that when an equation that includes values of both gastric pH and esophageal pH provides a significantly better fit of the data than an equation that includes corresponding values of gastric pH alone, we conclude that esophageal pH provides important information about values of gastric pH. Similarly, when an equation that includes values of both gastric pH and esophageal pH provides a significantly better fit of the data than an equation that includes corresponding values of esophageal pH alone, we conclude that gastric pH provides important information about values of esophageal pH. We have examined these possible relationships in pH recordings from normal subjects, GERD subjects and GERD + PPI subjects. As ingestion of meals alters both gastric pH and esophageal pH, we have analyzed data from entire 24‐h records as well as from daytime records when standard meals were ingested and nighttime records when subjects were fasting.

## Methods

The studies that generated the pH data used for the present analyses were approved by and conducted in compliance with good clinical practices as supervised by the Western Institutional Review Board, Olympia, WA. All subjects enrolled in these studies gave written informed consent. These studies were not registered with ClinicalTrials.gov because they were conducted in 2001 before the existence of this registry.

### Subjects

Normal subjects were 26 healthy adults with no history of gastrointestinal disease or symptoms who had not been treated with an investigational drug within 30 days prior to study entry. GERD subjects were 27 adults with a history of GERD who experienced heartburn at least four times per week for at least 6 months. These subjects had no history of a serious medical condition, other clinically significant gastrointestinal illness including peptic ulcer, difficulty swallowing thought to be due to a pathological process other than simple reflux, history of a gastrointestinal hemorrhage, upper GI surgery, stricture or esophageal dilation. Subjects had not been treated with an investigational drug within 30 days prior to study entry. Subjects had not used sucralfate, histamine H_2_‐receptor antagonists, misoprostol, proton pump inhibitors, promotility drugs or any other medication that alters gastric acid secretion or gastrointestinal motility within 1 week of baseline pH recording. Subjects had not used systemic corticosteroids, anticholinergics, antineoplastic agents, metoclopramide, anticoagulants, tetracycline or cisapride within 1 month of baseline pH recording. All normal and GERD subjects had a negative serology test for *Helicobacter pylori*.

### Study design

The study was conducted at the Oklahoma Foundation for Digestive Research on the campus of the University of Oklahoma Health Sciences Center. In normal subjects, 24‐h gastric pH and esophageal pH were measured on two separate occasions, 7 days apart. In GERD subjects, gastric pH and esophageal pH were measured for 24 h at baseline, and subjects were included in the study if esophageal pH ≤4 for at least 10% of the 24‐h recording period. These GERD subjects were then randomized to receive eight consecutive daily doses of 20 mg omeprazole or 20 mg rabeprazole in a cross‐over fashion with a 14‐day washout between treatment periods. Gastric pH and esophageal pH were measured for 24 h on days 1, 2, and 8 with each treatment. The present analyses of data from GERD subjects included baseline measurements and measurements on day 8 of treatment with omeprazole or rabeprazole.

All subjects fasted from approximately 22:00 the evening before until the beginning of pH recording the following morning at 8:00. Standardized meals were provided at breakfast (9:00), lunch (12:00) and dinner (18:00). Smoking and ingestion of food other than the test meals were prohibited during the pH recording periods.

Gastric and esophageal pH values were recorded every 4th second using an ambulatory, dual channel pH recording system (Medtronic Synectics, Shoreview, MN) with antimony electrodes. One electrode was placed in the stomach 10 cm below and the second was placed in the esophagus 5 cm above the manometrically defined upper border of the lower esophageal sphincter. Electrodes were calibrated to pH 1 and 7, and connected to a portable data storage unit (Digitrapper; Medtronic Synectics). Recordings began at 8:00 and continued for 24 h. Data were transferred from the portable data storage unit and processed using software designed for pH recordings (Polygram for Windows, Version 2.04; Medtronic Synectics).

The recordings used for the present analyses have been analyzed previously for other reports (Gardner et al. 2001, 2003a,b,c, 2004a,b).

### Analytical procedures

All pH values were adjusted for the temperature‐dependent variation in the relation between potential difference and pH as described previously (Gardner et al. 2006).

The 24‐h recordings were divided into daytime (beginning of recording until end of hour 14) and nighttime (beginning hour 15 until end of recording) periods. As reported previously (Gardner et al. 2003c), the duration of the daytime period was selected, because integrated gastric acidity over this period gave the optimal correlation with meal‐stimulated gastric acid secretion.

Vector autoregression was calculated using R programming language (Version 2.15, http://www.r-project.org). Values from each pH record were divided into sequential bins each of which contained the same number of pH values. Bin size varied from 1 pH value up to 10 consecutive pH values and the median was calculated for each bin.

The two nested models are defined as follows.

The simpler VAR model is given as
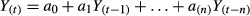


The complicated VAR model is given as
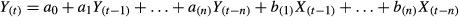


Where values of *Y* and *X* are medians from bins containing 1–10 consecutive pH values, *t* is time, and *n* is the lag that varies from 1 to 5. The magnitude of the lags and the bins in are always the same as in the simpler and complicated models. The value of the lag indicates the number of sequential bins and as the model contains one parameter for each sequential bin, the value of the lag also indicates the number of parameters in the model. As indicated by the equations above, at a given lag the complicated model always contains twice as many bins; and therefore, twice as many parameters as the simpler model. For example, at a lag of 2, if the simpler model contains two sequential bins for esophageal pH values with one parameter for each bin, the complicated model will contain the same two parameters for the same two bins of esophageal pH values as the simpler model plus 2 additional parameters for values from 2 corresponding bins of gastric pH values. Changing the lag changes the number of bins and parameters in the models; and therefore, the number of values being fit by the models but does not change the nature of the data being fit. Changing the bin size changes the nature of the data that are fit by the models in that the larger the bin the more sequential values that are used to calculate the median value for the bin. For example, a bin of 5 calculates the median of 5 sequential pH values, whereas a bin of 10 calculates the median of 10 sequential pH values. Fits of linear regressions of the two models to the data were compared using an *F*‐test for each pair of gastric and esophageal pH records. It is important to realize that VAR does not examine the fit of the simpler model alone or of the complicated model alone (Sims 1980). It only addresses whether the complicated model provides a significantly better fit of the data than the simpler model. In previous analyses (Gardner et al. 2005), however, we found that there is a statistical association of sequential esophageal pH values and of sequential gastric pH values for up to 2 h. This finding indicates that sequential pH values are tightly coupled in the simpler models used for the present analyses, and a given pH value provides important information regarding subsequent pH values.

When *Y* and *X* refer to gastric and esophageal pH, respectively, we label the relationship “E to G” to indicate information that values of esophageal pH provide regarding subsequent values of gastric pH. When *Y* and *X* refer to esophageal pH and gastric pH, respectively, we label the relationship “G to E” to indicate information that values of gastric pH provide regarding subsequent values of esophageal pH.

Using 133 24‐h records for gastric pH and esophageal pH, the raw data for the present analyses consisted of approximately 5.7 million pH values and the various fits using VAR included approximately 330 million values.

## Results

**Figure 1.**
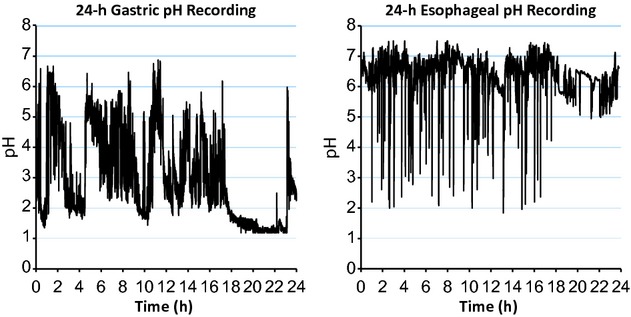
Representative 24‐h recording of gastric and esophageal pH from a normal healthy subject. Gastric and esophageal pH were recorded every 4th second for 24 h. Standard meals were ingested over 30 min at hours 1, 4 and 10.

Figure 1 illustrates a typical simultaneous recording of gastric pH and esophageal pH every 4th second for 24 h from a normal healthy subject. Each record consists of 21,600 values for gastric pH and 21,600 values for esophageal pH. Ingestion of a meal buffers intragastric acidity and increases gastric pH. The meal also stimulates gastric acid secretion which then causes gastric pH to decrease. There is obviously substantial variation in both gastric pH and esophageal pH over time, and visually there is no apparent relationship between individual values of gastric and esophageal pH. The main purpose of the present analyses was to explore the possibility that, values of esophageal pH can provide important information regarding subsequent values of gastric pH and vice versa.

**Figure 2.**
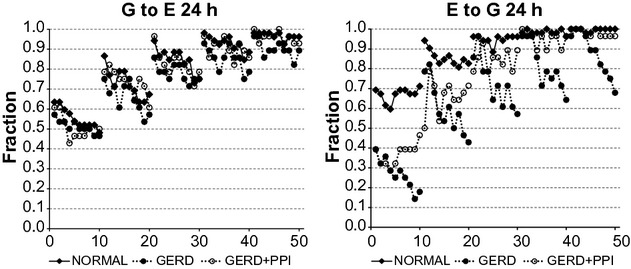
Fraction of 24‐h records from vector autoregression (VAR) for which the complicated model provides a significantly better fit of the data than the simpler model (*P* < 0.05 by *F*‐test). G to E refers to a comparison of VAR of esophageal pH alone to VAR of esophageal pH plus gastric pH. E to G refers to a comparison of VAR of gastric pH alone to VAR of gastric pH plus esophageal pH. Results are from 26 normal subjects and from 27 gastroesophageal reflux disease (GERD) subjects. On the *X* axis, values 1–10 indicate bins 1–10 with lag 1; values 11–20 indicate bins 1–10 with lag 2; values 21–30 indicate bins 1–10 with lag 3; values 31–40 indicate bins 1–10 with lag 4; and values 41–50 indicate bins 1–10 with lag 5.

Figure 2‐left illustrates that for G to E, results from normal records were essentially the same as those from GERD records and GERD + PPI records. For all groups of records, as the lag increased, there was an increase in the fraction of records for which the complicated model gave a significantly better fit than the simpler model (fraction of significant records). Also, at a given lag, as the bin size increased, there was a decrease in the fraction of significant records at lags 1–4 for normal records and at lags 1–3 for GERD and GERD + PPI records. These results indicate that values of gastric pH provide important information regarding subsequent values of esophageal pH and the nature of the relationship between values of gastric pH and subsequent values of esophageal pH is the same in normal and GERD subjects with or without PPI treatment.

Figure 2‐right illustrates that for E to G for all three groups of records, as the lag increased, there was a progressive increase in the fraction of significant records. For normal records, as the bin size increased, the fraction of significant records showed little or no change at all lags. For GERD records, however, as the bin size increased, the fraction of significant records showed a progressive decrease at all lags. Values for E to G from GERD + PPI records were between those from normal and GERD records at lags 1–3, and similar to those from normal records at lags 4 and 5. Furthermore, in GERD + PPI records, as the bin size increased there was little or no change in the fraction of significant records. These results in Figure 2‐right indicate that values of esophageal pH provide important information regarding subsequent values of gastric pH. In GERD subjects, however, this relationship decreases as the medians of more sequential pH values are considered. Treating GERD subjects with a PPI converts values for E to G toward those from normal subjects at lags 3–5 but not at lags 1 and 2.

**Figure 3.**
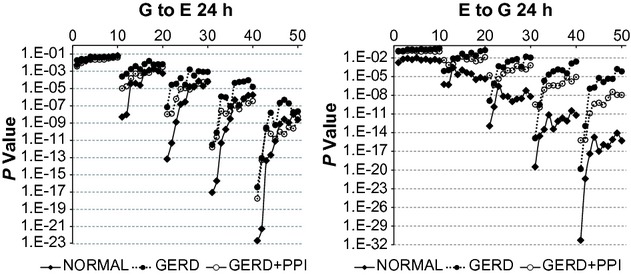
*P*‐values from *F*‐test for 24‐h records from vector autoregression (VAR) comparing the complicated model to the simpler model. G to E refers to a comparison of VAR of esophageal pH alone to VAR of esophageal pH plus gastric pH. E to G refers to a comparison of VAR of gastric pH alone to VAR of gastric pH plus esophageal pH. Results are medians from 26 normal subjects and from 27 gastroesophageal reflux disease (GERD) subjects. On the *X* axis, values 1–10 indicate bins 1–10 with lag 1; values 11–20 indicate bins 1–10 with lag 2; values 21–30 indicate bins 1–10 with lag 3; values 31–40 indicate bins 1–10 with lag 4; and values 41–50 indicate bins 1–10 with lag 5. Notice the different scales on the *Y*‐axes.

The results in Figure 2 give the fraction of records for which the complicated model gave a significantly better fit than the simpler model defined as an *F*‐test with *P* < 0.05. With a cutoff of *P* < 0.05 one could expect approximately 5% false‐positive results due to chance alone. To consider this possibility, we calculated the binomial probability of obtaining at least the fraction of significant records observed given the total number of records, number with *F*‐test giving *P* < 0.05 and a probability of 0.05. In Figure 3, all results from normal records and GERD + PPI records, as well as GERD records at lags 2–5 had a binomial probability <0.0001. For G to E for GERD records at lag 1, results with all bins had a binomial probability <0.0001. For E to G for GERD records at lag 1, results with bins 1–4 had a binomial probability <0.0001, but those from bins 5–10 had probabilities of 0.0002–0.0387.

Figure 2 illustrates that increasing the lag increased the fraction of significant records for both G to E and E to G. This phenomenon reflects the improved fit of the complicated model that results from adding parameters to and fitting more data by the regression equation. The similar patterns for the three groups of records for G to E reflect similarities in the abilities of values of gastric pH to provide information about subsequent values of esophageal pH. The different patterns among the three groups of records for E to G reflect differences between normal and GERD subjects with respect to the abilities of values of esophageal pH to provide information about subsequent values of gastric pH.

Figure 2 also illustrates that not all records showed that the complicated model provided a significantly better fit of the data than the simpler model, particularly at low lags with large bin size. This phenomenon may result from the skewed nature of the pH data within as well as between subjects coupled with the stochastic processes that give rise to gastric and esophageal pH. Figure 2 does illustrate, however, that for each group of subjects, with a complicated model with five added parameters (lag 5) and a bin size of 1 nearly all records show a significantly better fit with the complicated model compared to the simpler model.

The results in Figure 2 give the fraction of records for which the complicated model gave a significantly better fit of the data than the simpler model (*P* < 0.05 using an *F*‐test to compare the two linear regression models). The *P*‐value from the *F*‐test is also a measure of how much more information the complicated model provides regarding the data than the simpler model in that the lower the *P*‐value the greater the information provided by the complicated model. For example, a *P*‐value of 10^−6^ indicates that compared to the simpler model the complicated model provides more information regarding the data than does a *P*‐value of 10^−3^.

Figure 3‐left illustrates that for G to E from 24‐h records, *P*‐values for each group of records decreased progressively with increasing lag and at lags 2–5, *P*‐values at smaller bins were lower than those at larger bins. At lag 1, there were no changes in *P*‐values with increasing bin size and no important differences among results from normal, GERD or GERD + PPI records. For lags 2–5, at the smaller bins, *P*‐values were lower from normal records than from corresponding values from GERD or GERD + PPI records. At larger bins, *P*‐values were similar for normal, GERD, and GERD + PPI records.

Figure 3‐right illustrates that for E to G from 24‐h records, *P*‐values for each group of records decreased progressively with increasing lag and at lags 2–5, *P*‐values at smaller bins tended to be lower than those at larger bins. At all lags and bins, *P*‐values were lower from normal records than from corresponding values from GERD or GERD + PPI records. At lags 4 and 5, values from GERD + PPI records were between those from normal and GERD records.

The results in Figure 3 indicate that in normal, GERD and GERD + PPI records, values of gastric pH provide important information regarding subsequent values of esophageal pH and that values of esophageal pH provide important information regarding subsequent values of gastric pH. In both instances the magnitude of this information tends to vary directly with lag size and inversely with bin size. In addition, the magnitude of information provided by values of esophageal pH regarding subsequent values of gastric pH or vice versa is generally higher in normal records than in GERD records and higher than in GERD + PPI records at smaller bin sizes.

The increased information with increasing lag reflects an increase in the number of values fit by an increased number of parameters in the regression equations. For example, at lag 1, the complicated model contains one more parameter than the simpler model, whereas at lag 5 the complicated model contains five more parameters than the simpler model. As illustrated in Figure 3 for normal subjects, for G to E (Fig. 3‐left) at bin 1, lag 1, the *P*‐value is 3.6 × 10^−3^ and at bin 1, lag 5 is 2.3 × 10^−23^. For E to G (Fig. 3‐right) at bin 1, lag 1, the *P*‐value is 1.7 × 10^−3^ and at bin 1, lag 5 is 5.1 × 10^−32^. Thus, increasing the lag produces a substantial increase in the amount of information the complicated model provides regarding subsequent values of pH compared to the simpler model.

In Figure 3, *P*‐values for both G to E and E to G tended to be lower at smaller bin size, particularly at higher lags. These results indicate that the finer the granularity of the data, the greater the amount of information the complicated model provides regarding subsequent values of pH compared to the simpler model.

A change in the *P*‐value from an *F*‐test can result from a change in the fit of the simpler model, a change in the fit of the complicated model or a combination of the two. To examine these possibilities, we calculated the variance for VAR of the simpler model and the corresponding complicated model at different lags and bin sizes.

**Figure 4.**
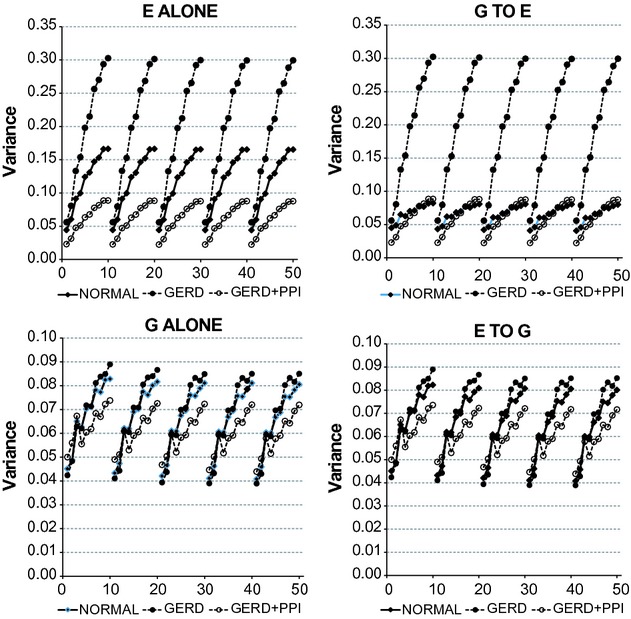
Values for variance for 24‐h records from vector autoregression (VAR) comparing the complicated model to the simpler model. E ALONE and G ALONE refer to VAR of the simpler model – esophageal pH alone and gastric pH alone, respectively. G to E and E TO G refer to VAR of the complicated model – esophageal pH plus gastric pH and gastric pH plus esophageal pH, respectively Results are medians from 26 normal subjects and from 27 gastroesophageal reflux disease (GERD) subjects. On the *X* axis, values 1–10 indicate bins 1–10 with lag 1; values 11–20 indicate bins 1–10 with lag 2; values 21–30 indicate bins 1–10 with lag 3; values 31–40 indicate bins 1–10 with lag 4; and values 41–50 indicate bins 1–10 with lag 5.

Figure 4 examines effects of changing lag and bin size on the fits of the data given by the simple models alone (E ALONE and G ALONE) and the complicated models alone (G TO E and E TO G). For both the simpler models and the complicated models, increasing bin size produced a progressive increase in the variance indicating a progressive decrease in the goodness of the fit of the model to the data. That is, decreasing the granularity of the data results in more information that cannot be accounted by the model parameters. In contrast, at a given bin size, increasing the lag produced little or no change in the variance. Except for E ALONE and G TO E from normal records, variances for the complicated model from all other records were the same as corresponding variances for the simpler model. For normal records variances for G TO E were lower than corresponding variances for E ALONE, but at a given bin size, increasing the lag produced little or no change in the variance. Thus, with the data for gastric and esophageal pH used for the present analyses, comparing variances from the complicated model to corresponding variances from the simpler model fails to detect the abilities of gastric pH to provide important information regarding subsequent values of esophageal pH and vice versa. In other words, analyzing the fits of the simpler model alone or of the complicated model alone provides information regarding effects of changing bin size and parameter number on the fits of the models to the data. They do not, however, address the major focus of the present analyses; namely, whether the complicated model provides a significantly better fit of the data than the simpler model, and if so, indicates that values of esophageal pH provide important information about subsequent values of gastric pH and vice versa.

The difference between results in Figures 3, 4 can be accounted for by differences in what is being calculated. The variance is calculated as the sum of the squares of the distance of a given value on the *Y*‐axis from the regression line (SS) divided by the degrees of freedom (df). The *F*‐test that was used for the data in Figures 2, 3 compares the relative change in SS going from the complicated model to the simpler model to the relative change in df in going from the complicated model to the simpler model.

In Figure 3, for both G TO E and E TO G, *P*‐values tended to be lower at smaller bins than at larger bins, particularly at higher lags indicating that the smaller the bin size, the better the complicated model fit the data compared to the simpler model. Figure 4 illustrates that for each group of records the variance increased with increasing bin size for both the simpler models as well as the complicated models and that with the exception of E ALONE and G TO E from normal records, variances for the complicated model from all other records were the same as corresponding variances for the simpler model. Increased variance with the simpler models would tend to decrease *P*‐values, whereas increased variance with the complicated models would tend to increase *P*‐values, The increased *P*‐values at larger bins illustrated in Figure 3 indicate that the poorer fits of the complicated models illustrated in Figure 4 outweigh the poorer fits of the simpler models in contributing to the *P*‐value. This may be difficult to appreciate from the values for variance given in Figure 4; however, even with the two smallest values for degrees of freedom (2150 and 2145 at lag 5, bin 10), the fractional difference in the sum‐of‐squares is multiplied by 429 in calculating the *F* value in the *F*‐test. Thus, VAR and having 21,600 pH values from each record makes it possible to identify relationships that might otherwise remain hidden if analyses were restricted to simply comparing variances.

Also in Figure 4, for each group of records, variances for G ALONE and G TO E were lower than corresponding variances for E ALONE and E TO G. These findings raise the possibility that ingestion of standard meals at prespecified times may have synchronized gastric pH and by so doing reduced the variances for both the simpler and complicated models.

The 24‐h period during which gastric and esophageal pH were recorded encompass two functionally distinct periods. The first 14 h of the recoding (daytime) was the period during which standard meals were ingested at prespecified times. The last 10 h of the recording (nighttime) was a fasting period. These two periods differ in terms of both gastric and esophageal acidity and in terms of values for a given measure of acidity from normal and GERD subjects (Kahrilas et al. 2013). For example, nighttime gastric acidity is typically higher than daytime gastric acidity. Over a 24‐h period and during nighttime, gastric acidity in normal subjects is not different from that in GERD subjects. During daytime, however, gastric acidity in GERD subjects is significantly higher than that in normal subjects. Esophageal acidity in GERD subjects is higher than that in normal subjects over 24 h as well as during daytime and nighttime. In view of these differences in acidity between daytime and nighttime periods, we examined the abilities of values of esophageal pH to provide important information about subsequent values of gastric pH and vice versa during daytime and nighttime periods.

**Figure 5.**
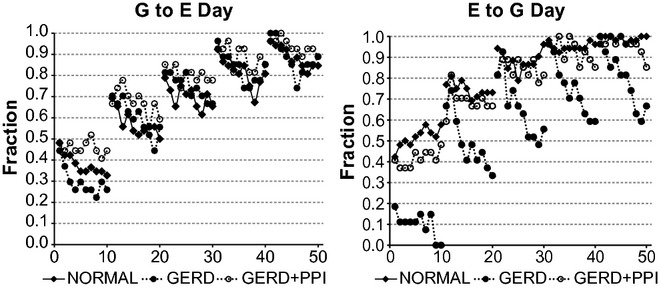
Fraction of daytime records from vector autoregression (VAR) for which the complicated model provides a significantly better fit of the data than the simpler model (*P* < 0.05 by *F*‐test). Daytime was from the beginning of the recording until the end of hour 14. G to E refers to a comparison of VAR of esophageal pH alone to VAR of esophageal pH plus gastric pH. E to G refers to a comparison of VAR of gastric pH alone to VAR of gastric pH plus esophageal pH. Results are from 26 normal subjects and from 27 gastroesophageal reflux disease (GERD) subjects. On the *X* axis, values 1–10 indicate bins 1–10 with lag 1; values 11–20 indicate bins 1–10 with lag 2; values 21–30 indicate bins 1–10 with lag 3; values 31–40 indicate bins 1–10 with lag 4; and values 41–50 indicate bins 1–10 with lag 5.

Figure 5 illustrates that results for the daytime fraction of significant records for G to E and E to G for normal, GERD and GERD + PPI records were similar to those during the entire 24‐h recording period illustrated in Figure 2. Also in Figure 5, all results for G to E from normal, GERD, and GERD + PPI subjects had a binomial probability <0.0001. All results for E to G from normal subjects and GERD + PPI subjects, as well as GERD subjects at lags 2–5 had a binomial probability <0.0001. For E to G from GERD subjects at lag 1, results with bins 1–10 had a binomial probabilities of 0.0085–0.7365.

**Figure 6.**
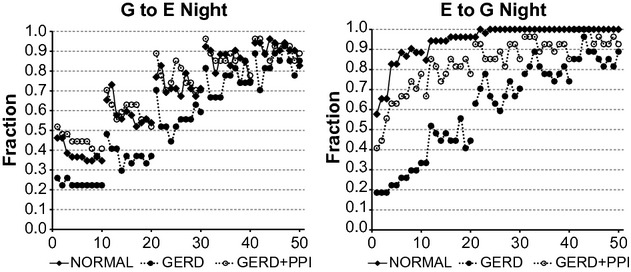
Fraction of nighttime records from vector autoregression (VAR) for which the complicated model provides a significantly better fit of the data than the simpler model (*P* < 0.05 by *F*‐test). Nighttime was from the beginning of hour 15 until the end of the recording. G to E refers to a comparison of VAR of esophageal pH alone to VAR of esophageal pH plus gastric pH. E to G refers to a comparison of VAR of gastric pH alone to VAR of gastric pH plus esophageal pH. Results are from 26 normal subjects and from 27 gastroesophageal reflux disease (GERD) subjects. On the *X* axis, values 1–10 indicate bins 1–10 with lag 1; values 11–20 indicate bins 1–10 with lag 2; values 21–30 indicate bins 1–10 with lag 3; values 31–40 indicate bins 1–10 with lag 4; and values 41–50 indicate bins 1–10 with lag 5.

Figure 6 illustrates that as was the case for daytime records (Fig. 5), for both G to E and E to G for each group of nighttime records, as the lag increased, there was a progressive increase in the fraction of significant records. As was also the case for daytime records, nighttime values for G to E from normal records were similar to those from GERD + PPI records. Values for G to E from GERD records, however, were generally below those from normal and GERD + PPI records.

Otherwise, however, nighttime values for both G to E and E to G (Fig. 6) showed a different pattern from corresponding values during daytime (Fig. 5). For G to E during daytime (Fig. 5‐left), as the bin size increased, there was a decrease in the fraction of significant records at all lags for all three groups of records. In contrast, for G to E during nighttime (Fig. 6‐left), as the bin size increased, there was a decrease in the fraction of significant records only for normal records at lags 1 and 2.

For E to G during daytime (Fig. 5‐right), as the bin size increased, the fraction of significant records did not change or increased for normal and GERD + PPI records, and decreased for GERD records. For E to G during nighttime (Fig. 6‐right), as the bin size increased, the fraction of significant records did not change or increased for each group of records.

In Figure 6, all results for G to E from normal, GERD, and GERD + PPI subjects had a binomial probability <0.0001. All results for E to G from normal subjects and GERD + PPI subjects, as well as GERD subjects at lags 2–5 had a binomial probability <0.0001. For E to G from GERD subjects at lag 1, results with bins 8–10 had a binomial probability <0.0001, but those from bins 1–7 had probabilities of 0.0002–0.0085.

**Figure 7.**
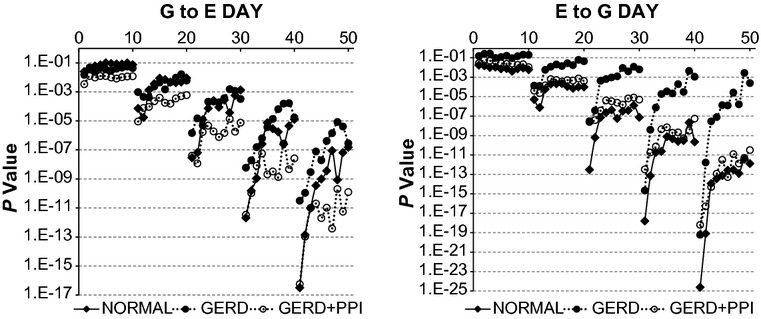
*P*‐values from *F*‐test for daytime records from vector autoregression (VAR) comparing the complicated model to the simpler model. G to E refers to a comparison of VAR of esophageal pH alone to VAR of esophageal pH plus gastric pH. E to G refers to a comparison of VAR of gastric pH alone to VAR of gastric pH plus esophageal pH. Results are medians from 26 normal subjects and from 27 gastroesophageal reflux disease (GERD) subjects. On the *X* axis, values 1–10 indicate bins 1–10 with lag 1; values 11–20 indicate bins 1–10 with lag 2; values 21–30 indicate bins 1–10 with lag 3; values 31–40 indicate bins 1–10 with lag 4; and values 41–50 indicate bins 1–10 with lag 5. Notice the different scales on the *Y*‐axes.

**Figure 8.**
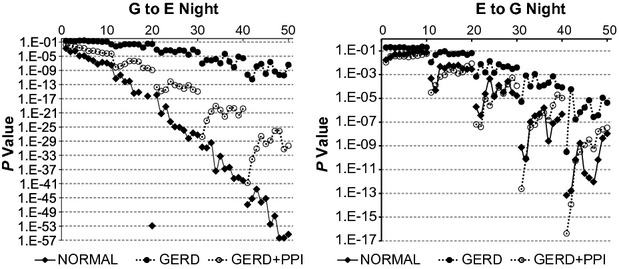
*P*‐values from *F*‐test for nighttime records from vector autoregression (VAR) comparing the complicated model to the simpler model. G to E refers to a comparison of VAR of esophageal pH alone to VAR of esophageal pH plus gastric pH. E to G refers to a comparison of VAR of gastric pH alone to VAR of gastric pH plus esophageal pH. Results are medians from 26 normal subjects and from 27 gastroesophageal reflux disease (GERD) subjects. On the *X* axis, values 1–10 indicate bins 1–10 with lag 1; values 11–20 indicate bins 1–10 with lag 2; values 21–30 indicate bins 1–10 with lag 3; values 31–40 indicate bins 1–10 with lag 4; and values 41–50 indicate bins 1–10 with lag 5. Notice the different scales on the *Y*‐axes and the outlier in the left panel.

As mentioned previously, the *P*‐value from the *F*‐test is a measure of how much more information the complicated model provides regarding the data than the simpler model. Figures 7, 8 illustrate *P*‐values during daytime and nighttime for the three groups of records and quantify the abilities of values of esophageal pH to provide important information about subsequent values of gastric pH and vice versa.

For G to E from daytime records, *P*‐values for each group of subjects decreased progressively with increasing lag and at lag 2–5, *P*‐values at smaller bins were higher than those at larger bins (Fig. 7‐left). At lag 1, *P*‐values did not change with increasing bin size and there were no important differences between results from normal and GERD records. At lag 1, values from GERD + PPI records were somewhat lower than those from normal or GERD records. For lags 2–5, at smaller bins, *P*‐values were generally lower from normal records than from corresponding values from GERD records but similar to those from GERD + PPI records. At larger bins, *P*‐values from normal records were similar to corresponding values from GERD records, and higher than those from GERD + PPI records.

For E to G from daytime records, *P*‐values for each group decreased progressively with increasing lag (Fig. 7‐right). Except at lag 1, *P*‐values at smaller bins tended to be lower than those at larger bins (Fig. 7‐right). At all lags and bins, *P*‐values were lower from normal records than from corresponding values from GERD records. *P*‐values from GERD + PPI records were slightly higher than corresponding values from normal records at smaller bins but similar to values from normal records at larger bins.

For G to E from nighttime records, *P*‐values for each group decreased progressively with increasing lag and the magnitude of the decrease was greatest with normal records, least with GERD records and intermediate with GERD + PPI records. (Fig. 8‐left). In contrast to corresponding daytime values (Fig. 7‐left) where values at larger bins tended to be higher than those at smaller bins, the opposite pattern occurred at nighttime; namely, *P*‐values from each group, except GERD + PPI at lags 4 and 5, tended to decrease or not change with increasing bin size. Nighttime *P*‐values for G to E from GERD records were higher than corresponding values from normal records and values from GERD + PPI were between values for these two groups. *P*‐values for G to E during nighttime (Fig. 8‐left) were consistently lower than corresponding values for G to E during daytime (Fig. 7‐left).

For each of the three groups, *P*‐values for E to G from nighttime records (Fig. 8‐right) showed a similar pattern to corresponding values from daytime records (Fig. 7‐right). *P*‐values for each group decreased progressively with increasing lag and except at lag 1 for GERD records, *P*‐values at larger bins tended to be higher than those at smaller bins. At all lags and bins, *P*‐values from normal records were lower than corresponding values from GERD records, but similar to corresponding values from GERD + PPI records.

Thus, as was the case with values for the fraction of significant records (Figs. 5, 6), *P*‐values in Figures 7, 8 illustrate that for each of the three groups of records, values of gastric pH and esophageal pH provide important information about subsequent values of esophageal pH and gastric pH, respectively. The magnitude of this information, however, is consistently lower in GERD records than in normal records. In addition, changes that occur with increasing bin size differ depending whether standard meals are being ingested or subjects are fasting, and these changes, in turn, reflect variations in the relationships between pH values over time.

## Discussion

There are two major findings from our analyses that are not apparent from visual inspection of pH tracings like those illustrated in Figure 1. First, values of esophageal pH provide important information about subsequent values of gastric pH and values of gastric pH provide important information about subsequent values of esophageal pH. These relationships hold for normal subjects, GERD subjects and GERD subjects treated with a PPI (in whom gastric acid secretion is reduced but not abolished). Furthermore, these relationships hold during daytime when meals are being ingested, during nighttime when subjects are fasting, and during the entire 24‐h period. Second, the ability of gastric pH to provide important information about subsequent values of esophageal pH and vice versa is clearly reduced in GERD compared to that in normal subjects.

The physiologic significance of gastroesophageal reflux is not known. Previously, however, we proposed a possible physiologic role for gastroesophageal reflux based on our finding a fractal pattern for both human gastric pH values and esophageal pH values (Gardner et al. 2005). The hypothesis that we proposed previously is that the fractal pattern encodes information regarding gastric acidity that is decoded by the esophagus during gastroesophageal reflux, and depending on the value of gastric acidity, the esophagus can signal the stomach to alter gastric acidity by altering gastric secretion of acid or bicarbonate (Gardner et al. 2005). At the present time, however, we do not have sufficient information to propose a mechanism other than to say that it could involve a neural, neuroimmunologic or hormonal influence. Previously, we also found that in pH records from normal subjects as well as GERD subjects or GERD subjects treated with a PPI, values of gastric pH could provide important information about subsequent values of gastric pH and that values of esophageal pH could provide important information about subsequent values of esophageal pH (Gardner et al. 2005). The present analyses were designed to test our hypothesis in the sense that this hypothesis predicts that values of gastric pH should provide important information about subsequent values of esophageal pH and values of esophageal pH should provide important information about subsequent values of gastric pH.

We addressed this possibility using VAR, a theory‐free set of inter‐related linear regressions used to measure relationships that can change over time. We found that using VAR to analyze pH records from normal subjects, GERD subjects and GERD subjects treated with a PPI during daytime, nighttime as well as the entire 24‐h period, gastric pH values provided important information regarding subsequent values of esophageal pH (G to E) and values of esophageal pH provided important information regarding subsequent values of gastric pH (E to G). These relationships held whether we measured the fraction of recordings for which the complicated model (values for gastric pH plus esophageal pH) provided a significantly better fit of the data than the simpler model (values for gastric pH alone or esophageal pH alone) or the *P*‐value from an *F*‐test comparing the fits of the two models. These results offer clear support for our hypothesis that depending on the value of gastric acidity, gastroesophageal reflux can cause the esophagus to signal the stomach to alter gastric secretion of acid or bicarbonate.

Nearly all investigators who measure esophageal pH or gastric pH for research purposes analyze summary values such as percent time pH ≤4 that are generated by commercially available software that is provided with the recording equipment. As with all summary values, the pH summaries have the advantage of communicating a large amount of data as simply as possible, for example, 21,600 values from a 24‐h pH recording are compressed to a single value for percent time pH ≤4. These summaries, however, do not consider possible relationships among individual pH values, and as a result, important relationships among these values may be hidden. Our previous analyses that used detrended fluctuation analyses to identify fractal patterns and lag analyses to show that individual pH values provided important information about subsequent pH values illustrate analyses can uncover hidden relationships among individual pH values (Gardner et al. 2005).

The present analyses extend our previous findings based on individual pH values and illustrate the important power of VAR to identify hidden relationships between constantly changing time‐series data for gastric pH and esophageal pH. We are unaware of other published analyses of possible relationships between time‐series data for gastric pH and esophageal pH besides our previous results (Gardner et al. 2005). One group (Clarke et al. 2009) has measured pH at 12 different locations from the distal esophagus to the proximal stomach over a 90‐min period to explore the region of low pH referred to as the “acid pocket” that occurs in the proximal stomach following ingestion of a meal (Kahrilas et al. 2013). These studies, however, did not evaluate individual pH values, but instead calculated the semiquantitative measure of time pH <4 over different recording intervals. Time‐series data from studies such as this, however, could possibly be analyzed using VAR to examine relationships among pH values recorded over time at one location and those recorded over time at another location.

As mentioned above under “Analytical procedures”, the size of the lag indicates the number of sequential bins in the models and the number of sequential values fit by the models. As each bin is associated with one parameter, the value of the lag also indicates the number of parameters in the model. At a given lag the complicated model always contains twice as many bins and twice as many parameters as the simpler model. In the present VAR analyses, we compared the fit of the complicated model to that of the simpler model by calculating the *P*‐value from an *F*‐test, and the critical issue was whether the complicated model provided a significantly better fit of the data than the simpler model.

Changing the size of the lag alters the number of parameters in as well as the number of values being fit by both the simpler model and the complicated model, but does not alter the nature of the data being fit by the models. We examined the effect of changing the size of the lag on the fraction of records for which the complicated model gave a significantly better fit of the data than the simpler model as well as on the *P*‐value from the *F*‐test. We found that for nearly all of the present results for G to E and E to G, increasing the lag increased the fraction of significant records and decreased the *P*‐value for which the complicated model gave a significantly better fit of the data than the simpler model for each group of records. Thus, considering more data by increasing the number of bins in the models amplifies the information that values of esophageal pH provide regarding subsequent values of gastric pH as well as the information that values of gastric pH provide regarding subsequent values of esophageal pH.

In contrast to changing the size of the lag, changing the size of the bins changes the number of values used to calculate the median in each bin and thereby changes the nature of the data being fit by the models. Changing the size of the bins does not alter the models themselves. For G to E and E to G during daytime and E to G during nighttime, *P*‐values at smaller bin sizes tended to be lower than corresponding values at larger bin sizes for each group of records. As increasing bin size decreases the granularity of the data, decreased granularity reduces the amount of important information that values of gastric pH provide regarding subsequent values of esophageal pH during daytime as well as the amount of important information that values of esophageal pH provide regarding subsequent values of gastric pH during both daytime and nighttime. In contrast, for G to E during nighttime, *P*‐values at smaller bin sizes tended to be higher than or the same as corresponding values at larger bin sizes for each group of records. Thus, decreasing the granularity of the data increases the amount of important information that values of gastric pH provide regarding subsequent values of esophageal pH during nighttime. These changes in information could have resulted from a change in noise or a change in the redundancy in the data being fit by the models (Gleick 2011). Decreasing noise or increasing redundancy in a system will increase information, whereas the opposite changes will decrease information (Gleick 2011).

It's conceivable, for example, that an increased *P*‐value with increasing bin size reflects decreased redundancy, whereas a decreased *P*‐value with increasing bin size reflects decreased noise. We cannot explain the different patterns for G to E or the similar patterns for E to G during daytime and nighttime other than to point out that daytime was characterized by ingestion of standard meals and marked changes in gastric pH, whereas nighttime was characterized by fasting and relative constant gastric pH.

The finding that decreasing the granularity of the data decreased the information that values of esophageal pH provide regarding subsequent values of gastric pH might explain our previous finding that infusing acid at a constant rate and concentration into the esophagus of normal subjects only reduced gastric acidity beginning 3 h after starting the infusion (Blondeau et al. 2009). The pH values of the infused acid would have had minimal variation that could have possibly limited its ability to influence gastric acidity.

When comparing two different sets of records in terms of *P*‐values from an *F*‐test or the fraction of records for which the complicated model gives a significantly better fit than the simpler model, differences can result from a difference between the fits of the simpler model, between fits of the complicated model or some combination of the two. For example, at a given lag and bin size, the median *P*‐value for GERD records could be higher than the corresponding median value from normal records because of a better fit of the simpler model to GERD data or a better fit of the complicated model to the normal data. Either difference, however, would indicate that compared to normal subjects, values of gastric pH in GERD subjects provide less important information about subsequent values of esophageal pH or vice versa. Previously, we found that esophageal pH values in GERD subjects were more tightly coupled statistically than corresponding values from normal subjects (Gardner et al. 2005). This tighter coupling in GERD subjects might limit the impact of information regarding values of esophageal pH on subsequent values of gastric pH and vice versa compared to their impact in normal subjects.

Our finding that the ability of gastric pH and esophageal pH to provide information regarding subsequent values of each other is clearly reduced in GERD records might indicate that an important defect in GERD is an impaired ability of changes in esophageal pH to trigger changes in gastric pH and vice versa. For example, esophageal acid exposure might fail to reduce gastric acid secretion and result in inappropriately elevated gastric acidity that, in turn, causes further esophageal acid exposure. Support for this possibility is our previous finding that meal‐stimulated gastric acid secretion is increased in GERD subjects compared to that in normal subjects (Gardner et al. 2003b). Such a phenomenon might result from an impaired ability of the esophagus to signal the stomach or alternatively, an impaired ability of the stomach to respond to inhibitory signals from the esophagus. Presently, however, we are unable to determine whether the decreased ability of gastric pH and esophageal pH to provide information regarding subsequent values of each other is a cause or a consequence of GERD.

Treating GERD subjects with a PPI shifts the overall patterns of fraction of significant records or *P*‐values from an *F*‐test from that observed in GERD records toward that observed in normal records. These findings indicate that changing gastric and/or esophageal acidity can change the abilities of gastric pH and esophageal pH to provide information regarding subsequent values of each other. We considered two possible explanations for these results. First, these findings could indicate that in GERD, the decreased ability of gastric and esophageal pH to provide important information about subsequent values of esophageal pH and gastric pH, respectively, is a consequence of some other underlying process that causes GERD. That is, the altered esophageal and gastric acidity in GERD in and of itself impairs the abilities of gastric pH and esophageal pH to provide important information regarding subsequent values of each other. Arguing against this possibility, however, is the difference between GERD and normal records during nighttime when there is no difference in integrated gastric acidity between these two groups during nighttime (Gardner et al. 2001, 2003c). Second, treating GERD subjects with a PPI may not simply reverse the primary abnormality in the relationships between sequential pH values observed in GERD records. For example, compared to normal records, the decreased ability of gastric pH to provide important information regarding subsequent values of esophageal pH and vice versa in GERD records might result from a poorer fit of the complicated model in GERD records. Compared to GERD records, however, the increased ability of gastric pH to provide important information regarding subsequent values of esophageal pH and vice versa in GERD + PPI records might result from a poorer fit of the simpler model with no change in the fit of the complicated model in GERD + PPI records. Such a phenomenon would shift the results toward those observed in normal subjects with no change in the fits of the complicated models.

The present analyses provide support for the role of the esophagus in influencing gastric acid secretion based on esophageal pH changes. The potentially protective physiology is teleologically consistent with physiologic homeostasis. The observation of impaired regulation in GERD provides an important hypothesis for the underlying pathophysiology and explains some of the difficulties encountered in clinical care of patients with GERD.

## Conflict of Interest

Luo Lu is currently employed at Twitter, Inc. Sheldon Sloan is an employee of Janssen Research and Development. Phillip B. Miner, Jr. is President and Medical Director of the Oklahoma Foundation for Digestive Research. Jerry D. Gardner is President of Science for Organizations, Inc. There is no conflict of interest financial or otherwise.
